# A retrospective study comparing holmium Laser enucleation vs. transurethral resection for the treatment of benign prostatic hyperplasia

**DOI:** 10.3389/fsurg.2026.1891814

**Published:** 2026-07-13

**Authors:** Zhikai Dong, Dongkai Wu, Zhongjie Tao

**Affiliations:** 1 Department of Urology, Dongyang People’s Hospital, Jinhua, Zhejiang, China; 2 Department of Urology, Pinghu Traditional Chinese Medicine Hospital, Jiaxing, Zhejiang, China

**Keywords:** benign prostatic hyperplasia, efficiency, holmium laser enucleation of the prostate, retrospective study, transurethral resection of the prostate

## Abstract

**Objective:**

Benign prostatic hyperplasia (BPH) is a common condition in aging men that significantly impacts quality of life. This study aimed to compare the perioperative outcomes and short-term efficacy of holmium laser enucleation of the prostate (HoLEP) vs. transurethral resection of the prostate (TURP) in patients with symptomatic BPH.

**Methods:**

We retrospectively analyzed data from 176 patients who underwent surgical treatment for BPH at our institution between January 2019 and December 2024. Patients were divided into two groups: HoLEP (*n* = 92) and TURP (*n* = 84). Baseline characteristics, operative parameters, postoperative complications, and functional outcomes in 6-month after surgery were compared between the groups.

**Results:**

Baseline demographics and prostate volume were similar between the two groups. The HoLEP group showed significantly shorter operation time (58.26 ± 20.11 min vs. 84.17 ± 36.80 min, *P* < 0.001), shorter catheterization time (5.37 ± 1.45 vs. 6.27 ± 1.47 days, *P* < 0.001), and reduced hospital stay (7.33 ± 3.12 vs. 13.61 ± 4.14 days, *P* < 0.001). Both groups demonstrated significant improvement in functional outcomes, but HoLEP had better international prostate symptom score, higher Qmax, (all *P* < 0.05). HoLEP had lower urinary tract irritation (22.62% vs. 42.86%) and erectile dysfunction (16.67% vs. 29.76%, both *P* < 0.05).

**Conclusion:**

HoLEP is a safe and effective alternative to TURP for BPH, offering advantages in perioperative safety and early recovery. It may be particularly beneficial in patients with large prostates or higher bleeding risk.

## 1 Introduction

Benign prostatic hyperplasia (BPH) is one of the most common urological conditions affecting aging men, with a prevalence that increases significantly after the age of 50 (1). Characterized by nonmalignant enlargement of the prostate gland, BPH can cause lower urinary tract symptoms such as frequency, urgency, nocturia, and weak urinary stream, all of which can significantly impair quality of life (2). When medical management with alpha-blockers or 5-alpha-reductase inhibitors fails to provide sufficient symptom relief, surgical intervention becomes necessary (3).

Transurethral resection of the prostate (TURP) has long been considered the gold standard surgical treatment for BPH (4). However, despite its effectiveness, TURP is associated with several limitations, including significant intraoperative bleeding, electrolyte imbalance, prolonged catheterization, and risks of TUR syndrome (5). These drawbacks have prompted the development of alternative minimally invasive surgical approaches.

Holmium laser enucleation of the prostate (HoLEP) is a relatively recent advancement in laser-based surgery (6). It utilizes a continuous-wave laser with a wavelength near the peak absorption of water, allowing for precise tissue vaporization, effective enucleation, and excellent hemostasis (7). Several studies have suggested that HoLEP may offer comparable, if not superior, functional outcomes to TURP, with a lower risk of perioperative complications and a shorter recovery period (8).

While numerous randomized controlled trials and meta-analyses have compared HoLEP and TURP, most existing research focuses on patients with large-volume prostates treated under standardized fast-track enhanced recovery protocols in high-volume specialized Western centers. Evidence remains limited regarding real-world comparative outcomes for elderly, multimorbid East Asian patients with BPH managed via conservative postoperative safety protocols at community hospitals. In this retrospective study, we aimed to compare the perioperative outcomes, postoperative functional recovery, and complication rates between HoLEP and conventional TURP in patients with symptomatic BPH. By evaluating real-world clinical data, we sought to provide further evidence on the safety and efficacy of Holmium laser technology as a viable alternative to traditional resection techniques.

## 2 Methods

### 2.1 Study design and patients

This was a non-randomized retrospective, observational, single-center cohort study aimed at comparing the clinical efficacy and safety of HoLEP vs. TURP in the treatment of BPH. We reviewed the electronic medical records of male patients who underwent either HoLEP or TURP for symptomatic BPH between January 1, 2019, and December 31, 2024. The choice between HoLEP and TURP depended on surgeon preference, equipment availability and comprehensive discussion with patients. No propensity score matching, multivariate confounding adjustment or other statistical balancing methods were applied to minimize selection bias, and only baseline demographic and clinical parameters were compared between groups to evaluate intergroup balance. The study was approved by the ethics committee of our hospital. All procedures were carried out in accordance with the principles of the Declaration of Helsinki.

### 2.2 Inclusion and exclusion criteria

Patients were eligible for inclusion when: (a) Male patients aged ≥ 50 years; (b) Diagnosed with symptomatic BPH based on clinical evaluation, digital rectal examination, and ultrasound examination; (c) Urodynamic examination revealed severe bladder outlet obstruction with maximum flow rate (Qmax) < 10 mL/s, good bladder detrusor tolerableness, and residual urine volume (RUV) ≥ 50 mL; (d) poor efficacy with conservative medical treatment for more than 1 year by international prostate symptom score (IPSS) and quality of life (QoL) evaluation.

The exclusion criteria were: (a) Prostate cancer; (b) History of previous prostate or urethral surgery; (c) Neurogenic bladder or significant detrusor underactivity; (d) Urethral stricture or bladder neck contracture; (e) Active urinary tract infection or hematuria of unknown cause; (f) Incomplete clinical or follow-up data.

Two attending urologists performed all operations, each with independent experience of over 100 HoLEP and bipolar TURP cases before 2019. No surgical protocol, laser/resectoscope equipment or operative technique was modified between January 2019 and December 2024, ensuring consistent surgical performance across the entire study timeframe.

### 2.3 Surgical procedures

All procedures were performed neuraxial anesthesia with the patient in the lithotomy position. In the TURP group, bipolar resection was performed in strict accordance with the 2018 Chinese Clinical Guideline for Bipolar Transurethral Resection of the Prostate and AUA surgical standards for BPH (9). A bipolar resectoscope (Olympus, 26 Fr) with a saline irrigation system (cutting power 160 W, coagulation power 80 W) was used. Two lateral incisions were made at the 5 and 7 o’clock positions extending from the bladder neck to the verumontanum to define the exenteration plane. Starting at the bladder neck, the prostate tissue was resected in chips from the median and lateral lobes toward the verumontanum. After tissue resection, resectoscope examination was performed to identify that there was no bleeding or residual tissue. At the end of the procedure, a 22F three-way Foley catheter was placed for bladder irrigation. Catheterization time and irrigation duration were based on intraoperative bleeding and individual patient recovery.

In the HoLEP group, the standardized three-lobe enucleation technique proposed by Gu et al. was adopted, following published technical updates for holmium laser prostate enucleation (10). The surgeons used a continuous-wave holmium laser (wavelength 2,140 nm) to enucleate of the prostate tissue followed by standard three-lobe enucleation technique. A 550-*μ*m laser fiber was introduced through the working channel of a laser-compatible endoscope. The energy output was set at 80 W (frequency 40 Hz, pulse energy 2 J), depending on the prostate volume and bleeding tendency. Power was increased to 90–100 W for rapid enucleation and enhanced hemostasis in prostates >60 mL with rich vascular tissue, while reduced power of 60–70 W was adopted for small glands (<40 mL) or thin capsular regions to minimize the risk of capsule perforation. All intraoperative power adjustments were fully recorded in surgical notes. Once exenteration was complete, the prostate tissue was pushed into the bladder and removed using a mechanical morcellator through the sheath.

Prophylactic intravenous antibiotics were administered 30 min prior to the procedure. A 26F resectoscope/enucleation scope was used in both groups for access to the bladder via the urethra.

Postoperative bladder irrigation was discontinued once the irrigation fluid became clear, and the catheter was removed thereafter according to a uniform institutional protocol that requires confirmation of clear urine over a 24-hour period before catheter removal.

### 2.4 Data collection

All data in this study were collected from electronic medical records. Preoperative variables including age, prostate volume, PSA level, baseline IPSS, QoL score, Qmax, and RUV. During the surgery, the operation time, estimated blood loss, weight of tissue resected, and intraoperative complications were recorded. The postoperative outcomes were catheterization time, hospital stay duration, complications (such as hematuria, clot retention, urinary retention, urinary tract infection), and functional outcomes at 3-, 6-month postoperatively. Furthermore, erectile dysfunction was evaluated using the IIEF-5 questionnaire at the 6-month follow-up; patients with a total IIEF-5 score ≤ 21 were classified as having erectile dysfunction. Urinary tract irritation was defined as persistent postoperative lower urinary tract symptoms including frequency, urgency, and dysuria requiring symptomatic treatment, excluding transient bladder discomfort directly induced by the indwelling catheter.

### 2.5 Statistical analysis

All analyses were performed using SPSS version 22.0 (IBM Corp., Armonk, NY). Normality of continuous variables was assessed using the Shapiro–Wilk test, and homogeneity of variance was evaluated using Levene's test. Normally distributed variables (including age, prostate volume, operation time, resected prostate weight, hemoglobin decline, irrigation time, catheterization time, hospital stay) with equal variances are presented as mean ± standard deviation (SD). Normally distributed data were presented as mean ± SD and compared between groups via independent samples t-test; non-normal data were presented as median (interquartile range) and compared via the Mann–Whitney U test. Functional outcomes (IPSS, QoL, Qmax, RUV) were used paired-samples t-test to analyze within-group changes from baseline, and independent samples t-test for intergroup comparisons. Categorical variables (retrograde ejaculation, urinary tract irritation, erectile dysfunction, postoperative bleeding, urinary tract infection, transient incontinence) are presented as frequencies and percentages, and were compared using the chi-square test or Fisher's exact test as appropriate. A two-sided *P* < 0.05 was considered statistically significant.

## 3 Results

### 3.1 Baseline characteristics

A total of 176 patients were included in the study, 92 patients in HoLEP group and 84 patients in TURP group. The mean age was 72.43 ± 7.68 years in the HoLEP group and 72.81 ± 6.99 years in the TURP group (*P* = 0.736). Preoperative prostate volume was 54.83 ± 21.2 ml in the HoLEP group and 54.69 ± 26.59 ml in the TURP group (*P* = 0.970). There were no statistically significant differences between the two groups in terms of baseline demographics and clinical parameters (Table 1).

**Table 1:** The baseline ncharacteristics of patients in two groups.

Group	HoLEP (*n* = 92)	TURP (*n* = 84)	P
Age/year	72.43 ± 7.68	72.81 ± 6.99	0.736
Prostate volume/ml	54.83 ± 21.20	54.69 ± 26.59	0.970
IPSS	25.22 ± 3.50	25.58 ± 4.01	0.519
QoL	4.72 ± 0.86	4.70 ± 0.85	0.907
Qmax (ml/s)	7.67 ± 3.83	7.76 ± 4.45	0.888
RUV (ml)	178.21 ± 185.89	176.62 ± 188.76	0.955
PSA (ng/ml)	4.04 ± 2.53	4.21 ± 3.82	0.725

HoLEP, holmium laser enucleation of the prostate; TURP, transurethral resection of the prostate; IPSS, international prostate symptom score; QoL, quality of life; Qmax, maximum flow rate; RUV, residual urine volume; PSA, prostate-specific antigen.

### 3.2 Perioperative outcomes

The HoLEP group showed significant advantages in multiple perioperative indicators compared with the TURP group. The operation time in the HoLEP group was shorter (58.26 ± 20.11 vs. 84.17 ± 36.80 min, *P* < 0.001), the resection weight of prostate was more (40.05 ± 17.46 vs. 30.51 ± 17.24 g, *P* < 0.001), and the decrease in hemoglobin was smaller (13.53 ± 6.66 vs. 16.67 ± 10.18 g/L, *P* = 0.018). Additionally, the HoLEP group had shorter postoperative irrigation time (1.32 ± 0.86 vs. 2.31 ± 0.85 days, *P* < 0.001), catheterization time (5.37 ± 1.45 vs. 6.27 ± 1.47 days, *P* < 0.001), and hospital stays (7.33 ± 3.12 vs. 13.61 ± 4.14 days, *P* < 0.001) (Table 2).

**Table 2:** Comparisons of perioperative indicators of patients in two groups.

Group	HoLEP (*n* = 92)	TURP (*n* = 84)	P
Operation time/min	58.26 ± 20.11	84.17 ± 36.80	<0.001
Prostate weight/g	40.05 ± 17.46	30.51 ± 17.24	<0.001
Hemoglobin decrease/g/dL	1.35 ± 0.67	1.67 ± 1.02	0.018
Postoperative irrigation time/d	1.32 ± 0.86	2.31 ± 0.85	<0.001
Catheterization time/d	5.37 ± 1.45	6.27 ± 1.47	<0.001
Hospital stays/d	7.33 ± 3.12	13.61 ± 4.14	<0.001

HoLEP, holmium laser enucleation of the prostate; TURP, transurethral resection of the prostate.

### 3.3 Clinical outcomes at follow-up

During the 6-month follow-ups, both groups showed significant improvement in IPSS, QoL, Qmax, and RUV compared to baseline (*P* < 0.001, Table 3). At 6 months postoperatively, the HoLEP group had lower IPSS scores than the TURP group (6.83 ± 2.13 vs. 7.63 ± 1.87, *P* = 0.009). The QoL in TURP group was lower than that in HoLEP group in the first follow up (1.39 ± 0.77 vs. 1.74 ± 0.66，*P* = 0.002), and recovered to the same level until 6 months after surgery (*P* = 0.475). The HoLEP group showed better performance in Qmax at 3 months postoperatively compared with the TURP group (*P* = 0.033). The HoLEP group had a lower RUV than the TURP group at 3 months postoperatively (*P* = 0.025).

**Table 3:** Clinical outcomes of patients in two groups.

Group	n	Baseline	3 months	6 months
IPSS score				
HoLEP	92	25.22 ± 3.50	7.08 ± 2.62	6.97 ± 2.18
TURP	84	25.58 ± 4.01	7.67 ± 2.42	7.63 ± 1.87
P		0.519	0.123	0.032
QoL score				
HoLEP	92	4.72 ± 0.86	1.34 ± 0.75	0.95 ± 0.65
TURP	84	4.70 ± 0.85	1.73 ± 0.66	1.05 ± 0.66
P		0.907	<0.001	0.303
Qmax/ml/s				
HoLEP	92	7.67 ± 3.83	24.77 ± 3.45	26.89 ± 7.23
TURP	84	7.76 ± 4.45	23.74 ± 2.87	24.60 ± 5.96
P		0.888	0.032	0.002
RUV/ml				
HoLEP	92	178.21 ± 185.89	14.10 ± 12.23	15.29 ± 4.75
TURP	84	176.62 ± 188.76	19.23 ± 17.11	18.81 ± 4.79
P		0.955	0.025	0.039

HoLEP, holmium laser enucleation of the prostate; TURP, transurethral resection of the prostate; IPSS, international prostate symptom score; QoL, quality of life; Qmax, maximum flow rate; RUV, residual urine volume.

### 3.4 Complications

The most common complications in both groups were retrograde ejaculation, urinary tract irritation, and erectile dysfunction (Table 4). The incidence of urinary tract irritation, and erectile dysfunction in HoLEP group was significantly lower than that in TURP group [22.62% vs. 42.86%, *P* = 0.005; 16.67% vs. 29.76%, (*P* = 0.044)]. Additionally, there were no significant difference in the incidences of bladder spasm, postoperative bleeding, urinary tract infection, or transient incontinence between the two groups (*P* > 0.05).

**Table 4:** Complications in two groups.

Group	HoLEP (*n* = 92)	TURP (*n* = 84)	P
Retrograde ejaculation	38 (41.30%)	41 (48.81%)	0.317
Urinary tract irritation	20 (21.74%)	36 (42.86%)	0.003
Erectile dysfunction	15 (16.30%)	25 (29.76%)	0.033
Bladder spasm	4 (4.35%)	10 (11.90%)	0.093
Postoperative bleeding	2 (2.17%)	5 (5.95%)	0.261
Urinary tract infection	1 (1.09%)	3 (3.57%)	0.349
Transient incontinence	1 (1.09%)	5 (5.95%)	0.105

HoLEP, holmium laser enucleation of the prostate; TURP, transurethral resection of the prostate.

## 4 Discussion

Per consensus definitions outlined in the 2023 AUA guideline and contemporary narrative reviews on BPH surgical management, minimally invasive surgical therapies (MISTs) for BPH refer to transurethral interventions that avoid abdominal wall incisions, minimize intraoperative hemorrhage, and shorten postoperative recovery relative to open simple prostatectomy (11). Both bipolar TURP and HoLEP fall under this MIST classification; unlike open surgery, both access the prostate solely via the natural urethral tract without cutaneous cuts (12). Among transurethral MISTs, HoLEP constitutes an advanced laser-assisted variant with documented superior hemostasis and faster early recovery compared to conventional bipolar TURP, consistent with published comparative meta-analyses (13).

Surgical technique selection should be based on multiple factors, including prostate size, surgeon expertise, equipment availability, and patient comorbidities (14). While TURP remains a widely accepted and effective approach, HoLEP is emerging as a strong alternative, especially in centers with access to laser technology and trained personnel (15). Additionally, HoLEP demonstrated clear advantages over TURP across multiple perioperative parameters, reflecting its technical efficiency and superior hemostatic capability (16, 17).

Patients received HoLEP experienced significantly shorter operative times, reduced hemoglobin loss, all of which contribute to decreased surgical trauma and improved perioperative stability (18). Furthermore, HoLEP patients had shorter postoperative irrigation duration, reduced catheterization time, and shorter hospital stays, indicating a faster postoperative recovery and earlier return to normal activity (19). These benefits are likely attributable to the laser's precise enucleation along the prostate capsule and its continuous-wave energy delivery, which provides excellent coagulation and minimizes bleeding (20, 21). The reduced perioperative burden observed with HoLEP may have important clinical implications, especially for elderly patients, those with comorbidities, or individuals at higher bleeding risk, and supports the growing preference for HoLEP as a minimally invasive yet effective alternative to traditional TURP (22). Notably, the recorded catheterization time and overall hospital stay in both groups appear longer than values reported in multiple published HoLEP and TURP studies implementing fast-track or day-surgery protocols (23, 24). This discrepancy stems from two key factors specific to our cohort and institutional practice. First, our study enrolled predominantly elderly patients (mean age >72 years), requiring prolonged surveillance for postoperative bleeding and voiding dysfunction. Second, our ward adheres to a conservative safety protocol: catheter removal was only permitted after 24 h of clear bladder irrigation fluid, and patients needed a complete successful void trial without gross hematuria before discharge, which is stricter than ERAS pathways adopted in high-volume specialized centers. Even under these restrictive local discharge standards, HoLEP still produced statistically meaningful reductions in irrigation, catheterization, and hospitalization length versus TURP, verifying its superior perioperative recovery profile within our real-world clinical setting.

Our findings align with robust evidence from randomized trials and meta-analyses, indicating that HoLEP yields superior long-term functional outcomes compared to TURP. Meta-analyses have shown that at 6–12 months postoperatively, patients in the HoLEP group achieved significantly better improvements in maximum urinary flow rate (Qmax) and International Prostate Symptom Score (IPSS) than those undergoing TURP (25). This may be attributed to the more complete removal of prostate tissue during enucleation, as well as the preservation of bladder neck function and reduced thermal injury compared to bipolar resection (26). In particular, the reduced residual tissue burden in HoLEP may contribute to lower rates of late obstruction and improved patient satisfaction. Moreover, large-scale RCTs with up to three years of follow-up have demonstrated that HoLEP maintains favorable symptom relief (AUA SS), enhanced post-void residual reduction, and equivalent Qmax compared to TURP, with similarly low rates of long-term complications and reoperation (27). These external findings reinforce our study's observation that HoLEP leads to slightly better functional outcomes at 3–6 months. Taken together, the compelling benefits of HoLEP in symptom relief, flow improvement, and sustained durability underscore its evolving status as a superior alternative to traditional TURP for the surgical management of BPH.

Importantly, our study found a lower but not statistically significant rate of early complications in the HoLEP group, except urinary tract irritation (22.62% vs. 42.86%, *P* = 0.005). Notably, one patient (1.19%) in the HoLEP group occurred postoperative bleeding, whereas the TURP group had a rate of 5.59% (*P* = 0.210). These findings highlight the superior hemostatic profile of holmium laser technology and its potential role in high-risk surgical candidates, including those with anticoagulation or large prostates (28).

This study delivers both confirmatory and novel real-world evidence to expand the existing literature comparing HoLEP and bipolar TURP. Consistent with previous randomized trials and meta-analyses, our single-center cohort reaffirms that HoLEP achieves superior perioperative hemostasis, shortened postoperative catheterization and hospitalization, and improved short-term urinary functional recovery. Our results demonstrate that HoLEP still delivers tangible recovery benefits even under strict safety-oriented postoperative management criteria, offering practical reference for community urology departments. However, despite the perioperative and short-term functional advantages of HoLEP observed in our cohort, this technique carries well-documented limitations that prevent it from being designated a universal first-line therapy for all patients with symptomatic BPH, and bipolar TURP retains an important, guideline-supported role in routine urologic practice. First, HoLEP has a steep learning curve requiring 25–50 supervised cases for surgeons to reach consistent proficiency; inadequate operator experience increases operative duration, bladder injury risk during morcellation, and postoperative irritative voiding symptoms, issues rarely encountered with standard TURP in trained hands. Second, HoLEP demands costly holmium laser hardware, which remains unavailable at many community hospitals, whereas bipolar TURP equipment is widely accessible with lower capital and maintenance costs. Third, meta-analyses have yielded inconsistent long-term functional comparisons: while HoLEP delivers clear perioperative hemostasis and shorter catheterization, several pooled analyses report no durable intergroup differences in IPSS, QoL or Qmax beyond 12 months, limiting the scope of its functional superiority claim.

Several limitations should be acknowledged. First, this retrospective non-randomized cohort inevitably carries residual selection bias, even though baseline indicators were well balanced; surgical allocation was determined by clinical practical factors rather than randomization, and no propensity score matching, weighting, or multivariate confounding adjustment was performed to eliminate unmeasured confounders such as long-term anticoagulation status and overall comorbidity severity. Second, follow-up was limited to 6 months, lacking long-term data on reoperation rates, sustained urinary function and late complications. Third, all surgeries were completed by surgeons with sufficient case volume, but subtle individual differences in surgical technique cannot be fully excluded. Fourth, this single-center study's postoperative recovery protocols (catheterization and hospital stay standards) may not be directly generalizable to other medical centers with different clinical pathways.

## 5 Conclusion

This single-center retrospective cohort compared HoLEP and bipolar TURP in elderly patients with moderate-volume BPH treated under conservative discharge protocols. Within our cohort, HoLEP produced significant short-term perioperative advantages: shorter surgery duration, less intraoperative blood loss, and briefer catheterization and hospitalization. At six months, HoLEP was associated with lower rates of IIEF-5-defined erectile dysfunction and urinary tract irritation. Superior IPSS and Qmax improvements were seen at three months, though intergroup functional gaps partially diminished by the six-month follow-up. Additional prospective, multicenter trials with extended follow-up are needed to confirm long-term comparative efficacy across wider BPH subgroups.

## Data Availability

The original contributions presented in the study are included in the article/Supplementary Material, further inquiries can be directed to the corresponding author.
